# 9.4 T small animal MRI using clinical components for direct translational studies

**DOI:** 10.1186/s12967-017-1373-7

**Published:** 2017-12-28

**Authors:** Jörg Felder, A. Avdo Celik, Chang-Hoon Choi, Stefan Schwan, N. Jon Shah

**Affiliations:** 1 Institute of Neuroscience and Medicine-4, Forschungszentrum Jülich, 52425 Jülich, Germany; 2 Institute of Neuroscience and Medicine-11, Forschungszentrum Jülich, 52425 Jülich, Germany; 3 Faculty of Medicine, Department of Neurology, JARA, RWTH Aachen University, 52074 Aachen, Germany

**Keywords:** High-field MRI, Preclinical MRI, Translational platform

## Abstract

**Background:**

Magnetic resonance is a major preclinical and clinical imaging modality ideally suited for longitudinal studies, e.g. in pharmacological developments. The lack of a proven platform that maintains an identical imaging protocol between preclinical and clinical platforms is solved with the construction of an animal scanner based on clinical hard- and software.

**Methods:**

A small animal magnet and gradient system were connected to a clinical MR system. Several hardware components were either modified or built in-house to achieve compatibility. The clinical software was modified to account for the different field-of-view of a preclinical MR system. The established scanner was evaluated using clinical QA protocols, and platform compatibility for translational research was verified against clinical scanners of different field strength.

**Results:**

The constructed animal scanner operates with the majority of clinical imaging sequences. Translational research is greatly facilitated as protocols can be shared between preclinical and clinical platforms. Hence, when maintaining sequences parameters, maximum similarity between pulses played out on a human or an animal system is maintained.

**Conclusion:**

Coupling of a small animal magnet with a clinical MR system is a flexible, easy to use way to establish and advance translational imaging capability. It provides cost and labor efficient translational capability as no tedious sequence reprogramming between moieties is required and cross-platform compatibility of sequences facilitates multi-center studies.

## Background

Translational research—which may be defined as: “studies that are designed to address human or animal diseases including development of drugs and treatments but excluding studies carried out for regulatory purposes” [1, 2]—accounts for a large proportion of animal studies carried out annually. The European Union reports that almost 19% of animals used in studies addressed research and development in the fields of human medicine, veterinary medicine and dentistry [3]. Mice and rats were the most widely used animals with 61 and 14%, respectively; studied mainly due to their relative ease of breeding and housing, their similar basic biology and chemistry with humans and the wide availability of excellently characterized genetically engineered strains [4].

Since the adoption of the three Rs (replacement, reduction, refinement) principle [5] into the European Directive 210/63/EU, medical imaging has become a key technique in translational research as “it provides a unique opportunity for studying disease from onset in real time, in a quantitative way and non-invasively. It is the preferred method to monitor disease progression and response to treatment in small-animal models in basic and preclinical science and acts as a bridge between novel discoveries and clinical implementation in patient treatment.” [6] Magnetic resonance imaging (MRI) is the second most frequently used modality in preclinical imaging, accounting for about 23% of all examinations [7].

While small rodent imaging can be carried out on human MRI scanners, dedicated small animal systems display performance benefits such as higher temporal and spatial resolution [8]. However, these dedicated systems usually operate with vendor specific software and require the reimplementation of MR sequences to facilitate translational imaging studies. In addition to the tedious reprogramming of the MRI sequences in another programming environment, this approach is also prone to creating mismatching sequences and consequently creating experimental discrepancies, which reduce translational validity. That this is in fact problematic is obvious from early reports on—e.g. compare [9] for a discussion on errors in T_2_ measurements due to different sampling strategies and differences in commercial implementations of multi-echo sequences. While there are currently only a few dedicated vendors for preclinical MRI systems, new companies are emerging, hoping to capitalize on the potential market. However, due to cost and complexity, advanced animal imaging sequences may not be readily available on all systems and will differ in their implementation. Shrinking the gap between preclinical and clinical studies, while changing experimental parameters as little as possible, makes transposition of data easier. Thus the development of a dedicated small animal MRI machine using clinical software presents a major step in bridging this gap for truly translational research.

According to Tsui et al. [10], combining a clinical MRI with a preclinical magnet is one way to advance translational research. Major applications envisaged for an imaging platform established in this way are MRI/MRS investigations that require extensive translational work. Most prominent areas are drug discovery, e.g. in tumor research [11], cardiovascular imaging [10] and imaging of the central nervous system [12, 13].

Here, the construction and operation of a unique 9.4 T MRI scanner for small rodents, which is based on a clinical system but connected to a preclinical magnet and gradient coils, is presented. It operates with clinical software and allows execution of sequences compiled for the analogous human MRI scanner family. An initial report of this work has been presented at the 24th International Conference of the Society of Magnetic Resonance in Medicine [14].

## Methods

**Fig. 1:**
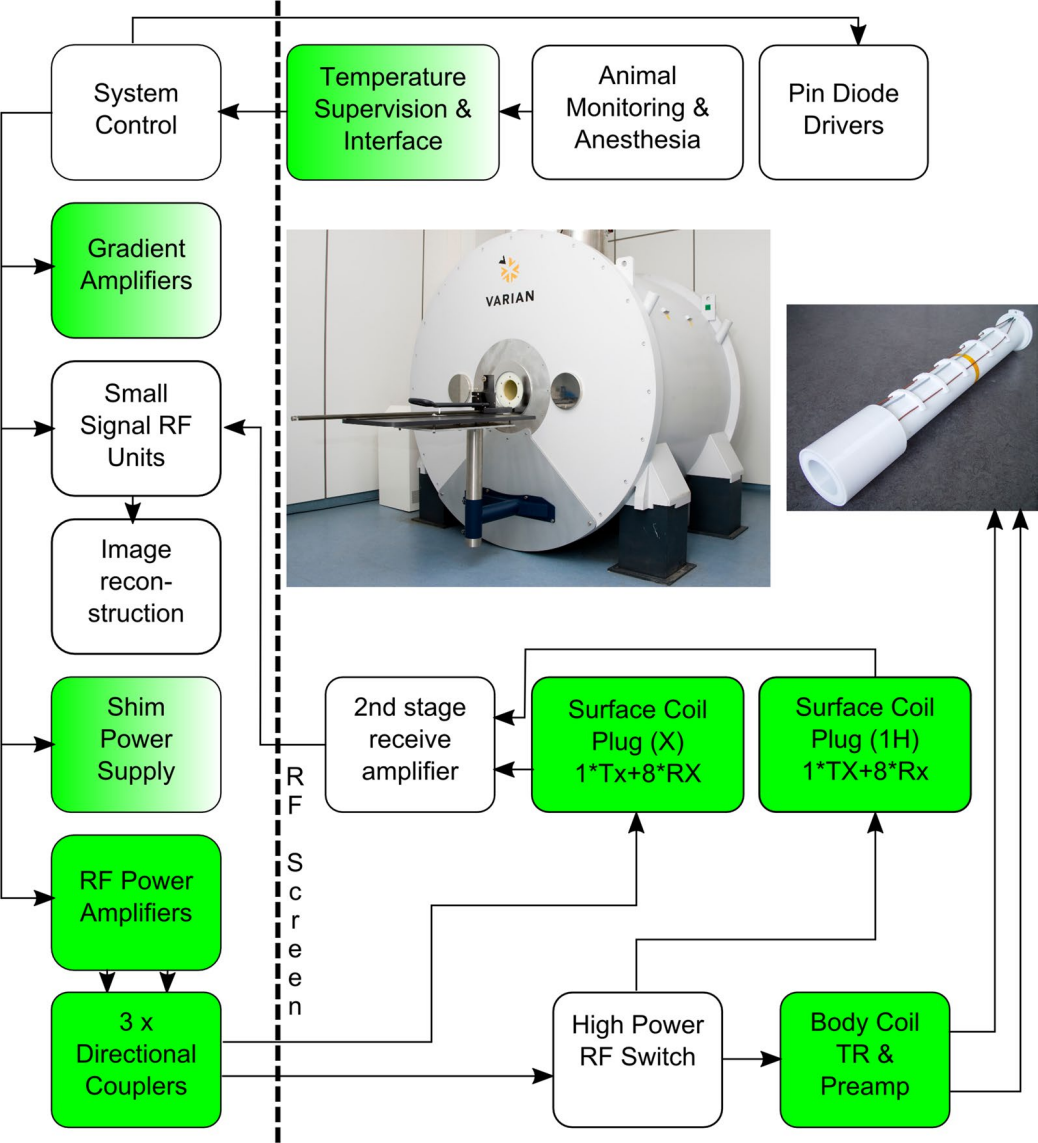
System overview showing major components and interconnections. In contrast to human high field systems this machine is equipped with a body coil. Specifically designed components have a green background, modified parts are marked with a color transition while untouched components have a while background

Figure 1 shows a system overview containing the major MR hardware components. The majority of electronic components are from a standard Trio, a Tim System (Siemens Healthcare GmbH, Erlangen, Germany). However, several modifications were required and these are described in more detail below. The user interface is based on the Syngo^®^ platform (Siemens Healthcare GmbH, Erlangen, Germany), and was also adapted to the modified imaging environment. The solution presented is based on modifying software parameters only and can potentially be upgraded to forthcoming new software baselines. Furthermore, it is capable of performing non-proton MR measurements, such as ^23^Na and ^31^P and is currently being extended to facilitated parallel transmission.

### Hardware components

#### Magnet

As in-house research takes place on a 9.4 T human MRI machine, in order to facilitate translational studies it was desirable that the same field strength be used for the animal system. A suitable 9.4 T 210 ASZ (Varian, Inc. Palo Alto, USA) magnet was sourced, to fit into an existing animal MRI suite. It has a free bore of 210 mm and was initially configured with a double cryostat filled with liquid helium and liquid nitrogen, respectively. However, it was retrospectively fitted with a pulse tube cryocooler (Cryomech, Inc. Syracuse, USA) that achieves zero boil-off operation and consequently only required that the nitrogen cryostat be filled with helium gas. The original magnet supervision and emergency discharge unit supplied by the magnet vendor was maintained without integrating these into the clinical system. Although integration is technically feasible, the interface between the different supervision units is complex and would not significantly alleviate system handling.

#### Gradient and shim system

**Fig. 2:**
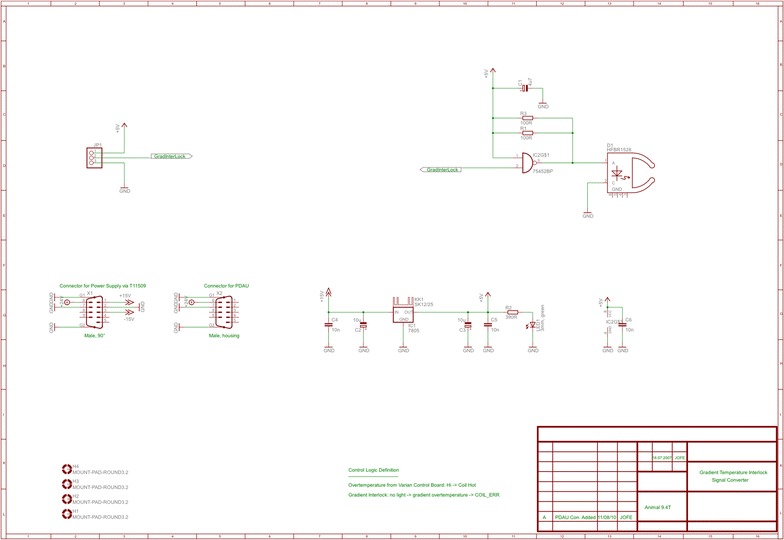
Circuit diagram of gradient interlock interface

In addition to the inherent demand for high gradient strength and slew rate, the requirements for the gradient insert are mechanical compatibility with the magnet dimensions; an inner diameter suitable for measuring small animals, e.g. rats and mice; and, importantly, compatibility with the clinical gradient amplifiers. Compatibility with the clinical gradient amplifiers was found to be essential, as the gradient amplifiers of a standard clinical system are designed for the strong drive requirements of large volume human gradient coils. However, by disabling single stages of the multiple-stage H-bridge amplifier configuration, it was possible to reduce the maximum output power of the gradient amplifiers. Following these modifications, the current output capability of each gradient amplifier was reduced to a maximum of approx. 320 A—disabling the appropriate amplifier stages can be achieved by jumpers on the control circuitry—making them compatible with commercially available gradient inserts such as the BGA12S (Bruker Cooperation, Billerica, USA) or the SGRAD 205/120/HD/S (Varian, Inc. Palo Alto, USA). The later was chosen for the system described here. Further hardware modifications of the gradient amplifier system were made on the ohmic loss supervision and the maximum pulse length supervision, depending on the applied output current. This was accomplished by modifying the analogue supervision circuitry of the gradient amplifiers and required changing resistor values in the operational amplifier based integration circuits. In addition, the temperature of the gradient insert is monitored using integrated PT100 temperature sensors and a vendor supplied monitoring circuit connected to a novel interface unit that sends a temperature interlock signal to the clinical MR system (Fig. 2). As an additional safeguard, gas discharge units were connected in parallel to the gradient coils in order to protect them in case the gradient amplifiers exceeded the maximum voltage settings (e.g. in case of an emergency electric shutdown).

In the system used, the shim coils integrated into the gradient insert are driven by a standard power supply MXH-5-CO (Resonance Research Inc., Billerica, USA). Each of the five current sources has the capability to drive ± 10 A into the shim coils. A vendor supplied CAN-bus (Controller Area Network—a standardised serial bus system to reduce wire count) to serial interface was used to connect the shim amplifiers to the MR scanner.

#### RF chain and coils

The RF power amplifiers (barthel HF-Technik GmbH, Aachen, Germany) were specifically designed for the platform described here and consist of a narrowband amplifier for protons and a broadband amplifier for X-nuclei, each capable of delivering 1 kW peak power. The proton amplifier combines the output power of four independent 250 W units—a design option chosen to facilitate later extension of the system for parallel transmit operation. The X-nucleus amplifier covers a frequency range from 50 to 305 MHz, which allows imaging of the most biologically relevant nuclei, such as ^13^C, ^17^O, ^23^Na and ^31^P. It is important to note that the proton amplifier also covers the ^19^F frequency of approx. 376 MHz at 9.4 T, albeit with a slightly reduced peak output power. Both power amplifiers were integrated into the clinical MR platform through a CAN bus interface, which was developed by the manufacturer of the amplifiers.

**Fig. 3:**
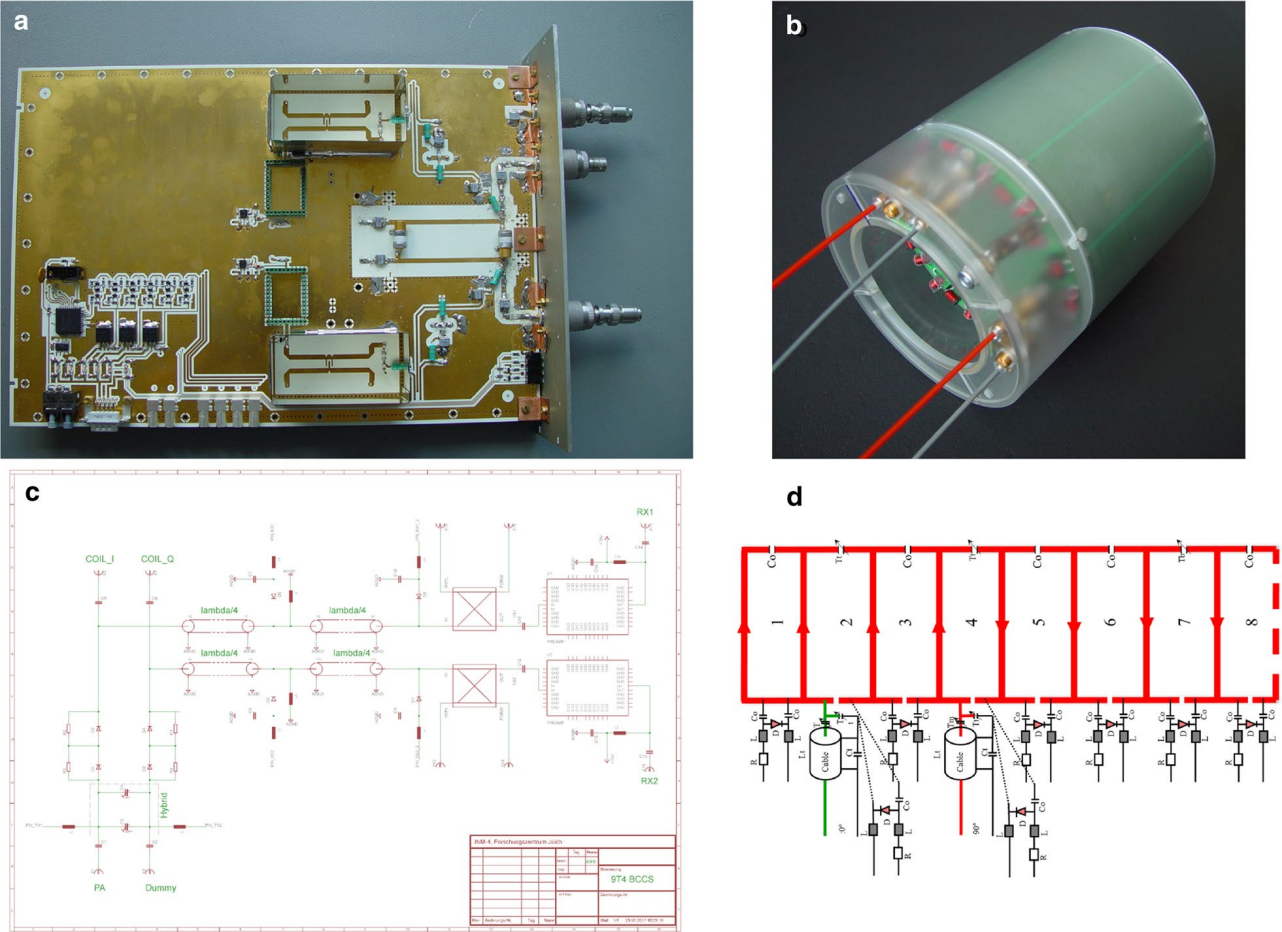
Major home built RF components: **a** T/R unit for the body coil containing printed quadrature hybrid, directional couplers in the receive path, preamplifiers as well as logic and drive circuitry to control the PIN diode bias. The unit is shown here without the RF enclosure, **b** Prototype body coil (8-rung birdcage), **c** schematic showing the RF circuit of the body coil T/R unit and **d** circuit diagram of the body coil including PIN diodes for detuning

In contrast to human high field scanners, the animal system is equipped with a body-coil. This conventional system setup is appropriate since wavelength effects in small animal systems are less pronounced due to the smaller size of imaged objects. Consequently, it was necessary to develop both the body-coil as well as the corresponding transmit/receive (T/R) switch in house. The T/R unit consists of a transmit quadrature-hybrid, directional couplers for power supervision, PIN diode based T/R switches as well as low noise preamplifiers for the 0° and 90° path, respectively (Fig. 3a, c). The body-coil is a self-shielded, 8-rung, high-pass quadrature birdcage with a free inner diameter of 74 mm (Fig. 3b, d). Capacitors are 4.7 pF series 25 type (Voltronics Corp., Cazenovia, USA), trimmers for tuning, matching and balancing use 25 pF NMA_HW series from the same manufacturer and PIN diodes employed are DH80106-11 N (Cobham plc, Dorset, UK.). It is possible to operate the body-coil either in T/R mode or in transmit (TX) only mode by detuning it with PIN diodes and receiving with local receive only coils. It can also be detuned statically in case local transmitter coils are used for excitation.

Local coils, e.g. surface coil arrays, can be connected via two dedicated coil connectors—using the ODU MAC non-magnetic connector series (ODU GmbH & Co. KG, Mühldorf, Germany)—one on the front and one at the back of the magnet. Each connector supports up to eight receive channels with the front connector being equipped with a proton transmit and the back connector with an additional X transmit channel. The available X-nucleus imaging coil set has been described previously [15]. All coils are detected by their unique coil code and the system automatically sets PIN diode control and sequence parameters according to the connected coil systems, as known from clinical routine.

#### Animal handling and monitoring

Animal handling includes the table shown in Fig. 1 (Bruker Cooperation, Billerica, USA), which is mechanically interfaced to a rat or mouse animal bed (RAPID Biomedical GmbH, Rimpar, Germany) allowing the animal to be manually slid into the magnet. The animal bed includes a facemask for gas anesthesia, which is connected to a standard veterinarian vaporizer (A.M. Bickford, Inc., New York, USA) and a charcoal filter to remove unused anesthetic.

Animal supervision, allowing the acquisition of ECG, respiration and temperature, uses a commercially available monitoring system (Small Animal Instruments, Inc., New York, USA). The system provides a user configurable trigger signal that is interfaced to the MRI scanner to enable synchronized sequences using the ECG or breathing signals of the animal.

### Software modifications

Due to both its use as a translational platform and the complexities involved in reprogramming, an important requirement of the animal MR scanner is that it can be operated with only minor tweaks to the measurement parameters and without having to modify major parts of its software. Ordinarily two major modifications would be required relating to field-of-view (FoV) settings of an animal system compared to a human scanner and hardware component differences. The latter was addressed by disabling software supervision of non-existing components, e.g. the patient bed. FoV settings were amended by introducing a 1:5 scaling of linear dimensions. By doing this, the FoV discrepancy was reduced to an extent that the clinical software worked with the smaller imaging dimensions of the animal system. The scaling was implemented by modifying gradient and shim sensitivities accordingly.

In addition, the system tune-up also required several parameter modifications. The most prominent being, definition of tune-up phantoms used with the animal system, modification of hardware supervision such as minimum flow of cooling water through the gradient insert, and a reduction of the transmit power limits to account for the less potential power amplifies in an animal environment. Modification of parameters is carried out by changing these in the measurement settings file of the clinical MR software.

### Experiments

Several experiments were carried out after establishing system validity based on clinical quality assurance (QA) routines to validate the translational workflow. In all cases, compiled sequences were copied on both systems and data acquired using a clinical scanner as well as the animal platform is presented here. For comparative purposes, the basic MR sequences—spin-echo and gradient-echo—were employed as they are the building blocks for all advanced imaging sequences. All adjustments (frequency, reference TX power and shimming) were carried out using the system’s automated workflow.

A set of phantom images was acquired using a standard spin echo sequence with isotropic resolution. Details of the imaging parameters are shown in Table 1. The phantoms employed were a 170 mm diameter spherical plastic phantom filled with doped water (1.25 g NiSO_4_ + 5 g NaCl per 1000 g distilled water) having a T_1_ of approximately 350 ms at 3 T and an animal sized phantom (T_1_ ≈ 560 ms) of 40 mm diameter (0.0444 g MnCl_2_ + 0.0667 g NaCl per 1000 g distilled water) for 9.4 T. For this sequence, small signal RF output as well as the D/A converter output of the three gradient axes were monitored with an oscilloscope (Tektronix, Inc., Beaverton, USA). All images were acquired with the body-coil of the respective MRI system.

**Table 1:** Sequence parameters for the different experiments

Sequence (System)	TR (ms)	TE (ms)	Slice (mm)	Matrix FoV (mm)	BW (Hz/Pixel)	FA (deg.)	Coil	
SE (9.4 T Animal)	1000	10	0.5	128 × 128 64 × 64	221	90	Body-boil	Phantom images (Figure 4)
SE (3 T Human)	1000	10	2.5	128 × 128 320 × 320	221	90	Body-coil	
GRE (9.4 T Animal)	95	10	1	256 × 256 50 × 50	260	25	Body-coil	Brain images (Figure 5)
GRE (9.4 T Human)	95	10	2	256 × 256 250 × 250	260	25	8ch pTX	
TSE (9.4 T Animal)	850	7	0.7	384 × 384 60 × 60	260	140	Body-coil + 2 channel RX	Spinal cord (Figure 6)
EPI (9.4 T Animal)	3000	6.2	1	64 × 64 60 × 60	4882	35	Body coil	EPI (Figure 7)
Multi-GRE (9.4 T Animal)	75	2.5, 5, 7.5, 10, 12,5	1	64 × 64 60 × 60	500	7	Body coil	B0 hom. (Figure 8)
AFI (9.4 T Animal)	750	2.72	1	64 × 64 (60 × 60)	330	60	Body coil	B1 hom. (Figure 8)

As a second test-case, images were also acquired with the vendor supplied gradient echo sequence at 9.4 T. Sequence parameters for the acquisition at the same field strength were kept identical, except for resolution and slice thickness, which were adapted to the desired FoV (compare Table 1). Due to the wavelength effect associated with the larger dimensions of the human body the 9.4 T human head acquisitions made use of a B_1_^+^ shimming approach [16]. A home-built 8-channel transceiver array was used as T/R coil [17] rather than the quadrature birdcage employed for the rat measurement.

Advanced imaging experiments were carried out on the animal scanner only to validate its performance using state of the art MR sequences. The first investigation images a rat spinal cord using a turbo-spin-echo sequence with gating on the animal breathing. The sequence employed is derived from a clinical T_1_ weighted spinal cord sequence. Modifications required for 9.4 T small animal imaging were different echo- and repetition time to account for the different field strength, an adaption of the FoV to the animal dimensions and a change of turbo-factor to allow gated acquisition without concatenations. The second analysis uses an echo planar readout for a single shot acquisition of the 40 mm diameter phantom described above to demonstrate gradient performance in the scope of combining clinical and non-clinical hardware. The EPI sequence was derived from a clinical sequence supplied by the vendor and sequence parameters optimized for the small animal system (compare Table 1).

Finally, quantitative performance measures were acquired to evaluate static and RF field homogeneity of the system. Based on the principles described in [18] B_0_ field maps were generated. The field map data was acquired using a standard multi-echo GRE sequence. Constant phase offsets were removed by subtracting the first echo from all following echoes. Resulting phase difference images were spatially unwrapped using FSL PRELUDE (FMRIB, Oxford, UK) [19]. Quantitative fieldmaps were estimated by linear regression of the unwrapped signal phase difference versus echo time difference. The actual flip angle method as implemented in [20] was used for plotting the flip angle distribution of the animal body coil.

## Results

### QA measurements

To establish initial operation and in order to perform a “sanity check” the clinical QA protocols of the MRI system were successfully carried out. They include: image orientation, RF amplifier linearity, phantom shimming, gradient cross term compensation, eddy current compensation, gradient delay compensation, gradient sensitivity, image artefact, fat saturation and short term field drift. The long-term field stability test was not carried out as the same magnet had already operated with different scanner hardware prior to being transformed into a translational system and there were no issues with field drifts.

### Translational sequence measurement

**Fig. 4:**
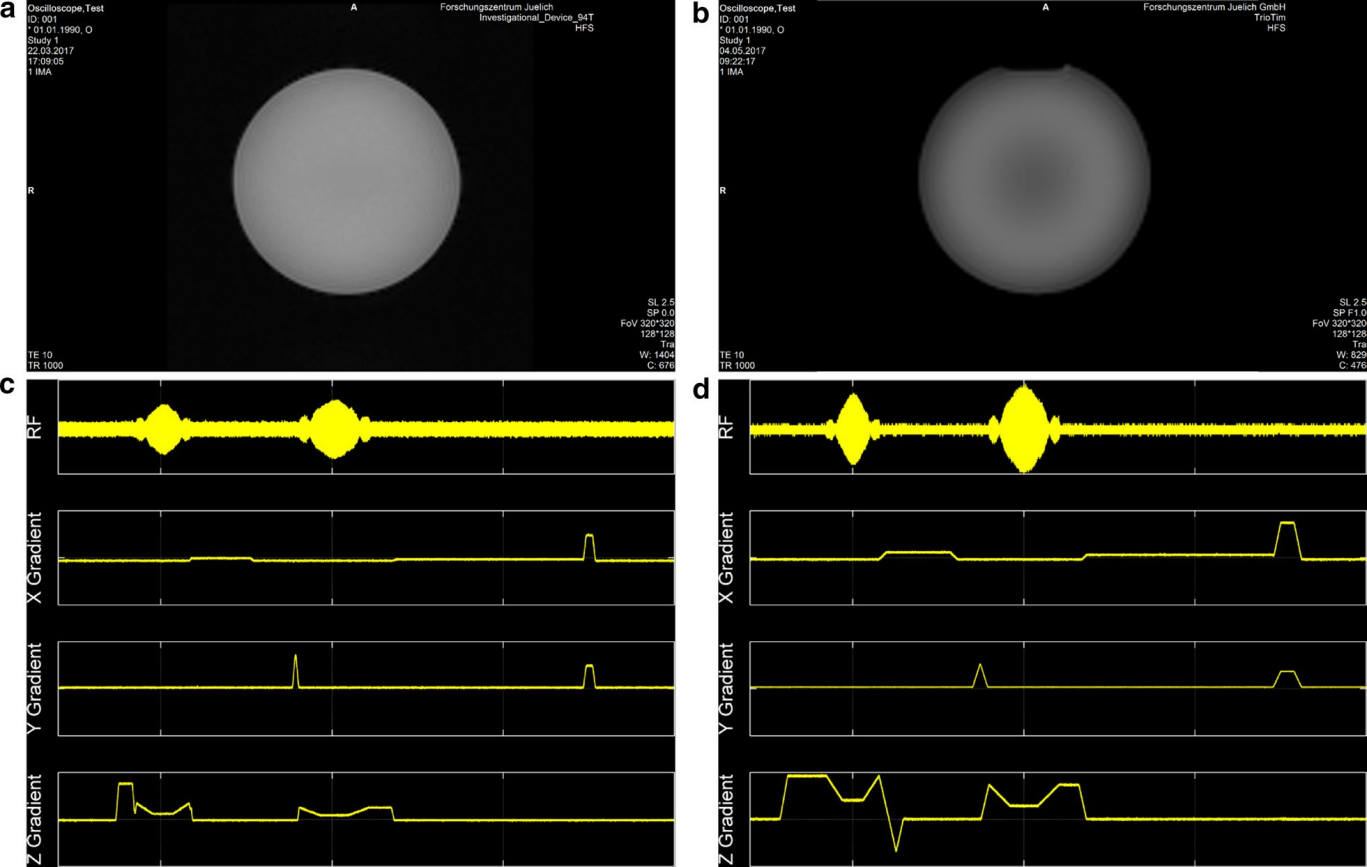
MR images and corresponding oscilloscope traces acquired with a vendor supplied spin echo sequence: **a** animal scanner phantom image, **b** human scanner phantom image. Pulse sequence timing diagram measured **c** at the animal scanner and **d** at the human scanner

Phantom images, along with the acquired pulse sequence traces for the first k-space line are shown in Fig. 4. Both images show the homogeneous contrast distribution that is expected for single compartment phantoms. The 3 T phantom image, however, displays some remaining non-uniformity in image intensity. The sequence diagrams directly measured using an oscilloscope show identical timing observable through same locations of excitation pulses (same TE), same RF excitation pulse envelope, and similar gradient waveform and crusher gradients.

### In vivo translational imaging

**Fig. 5:**
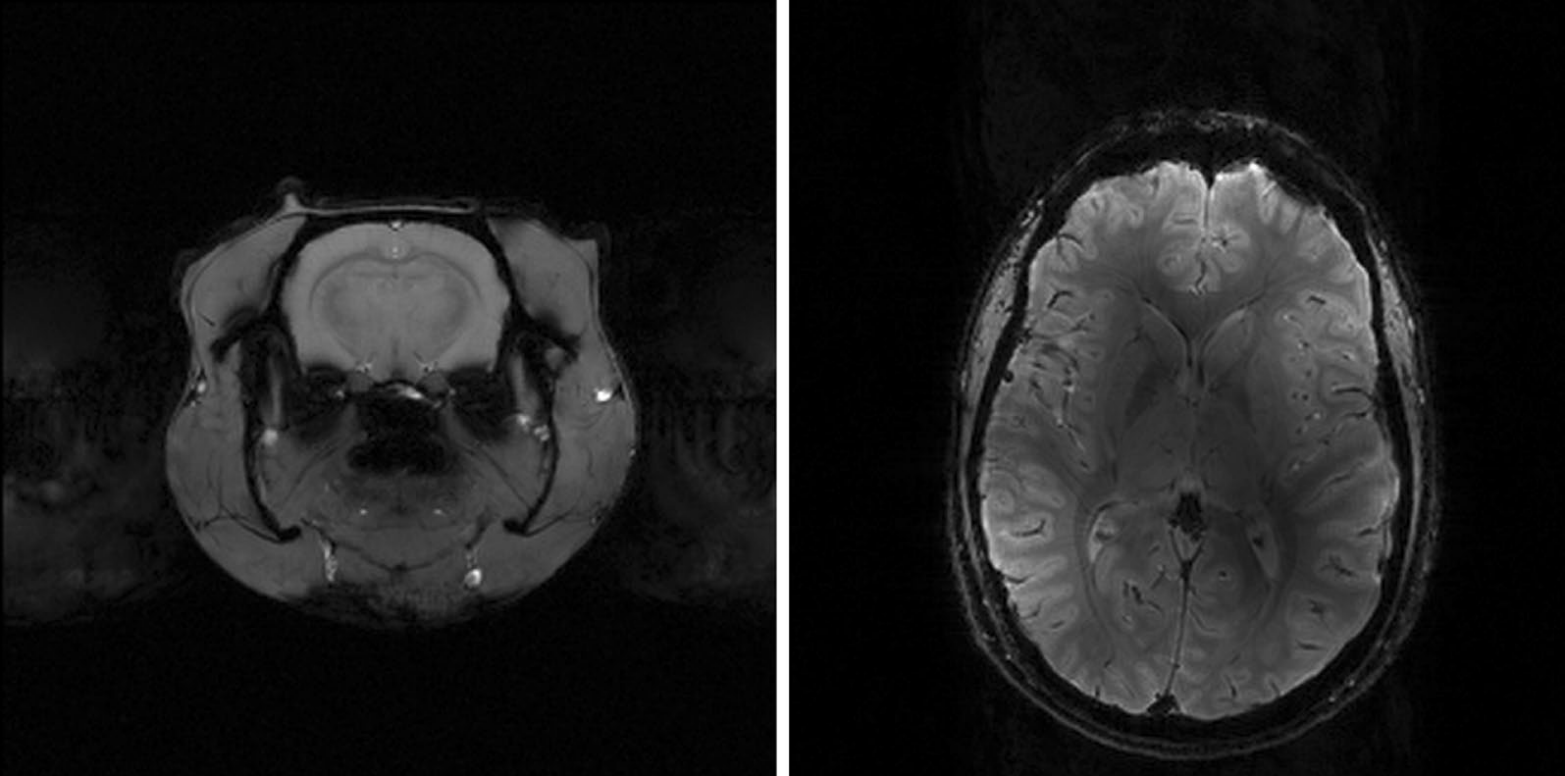
Single slice gradient echo images acquired with the vendor supplied sequence using identical measurement parameters: rat brain image from the 9.4 T animal system (left) and brain of volunteer from 9.4 T human scanner (right)

Figure 5 shows a single axial slice of a rat brain and a healthy human volunteer brain obtained using a multi-slice gradient recalled echo sequence, with the translational platform of two 9.4 T MRI systems—the animal system described here and a 9.4 T human system.

### Exemplary investigation: rat spinal cord

**Fig. 6:**
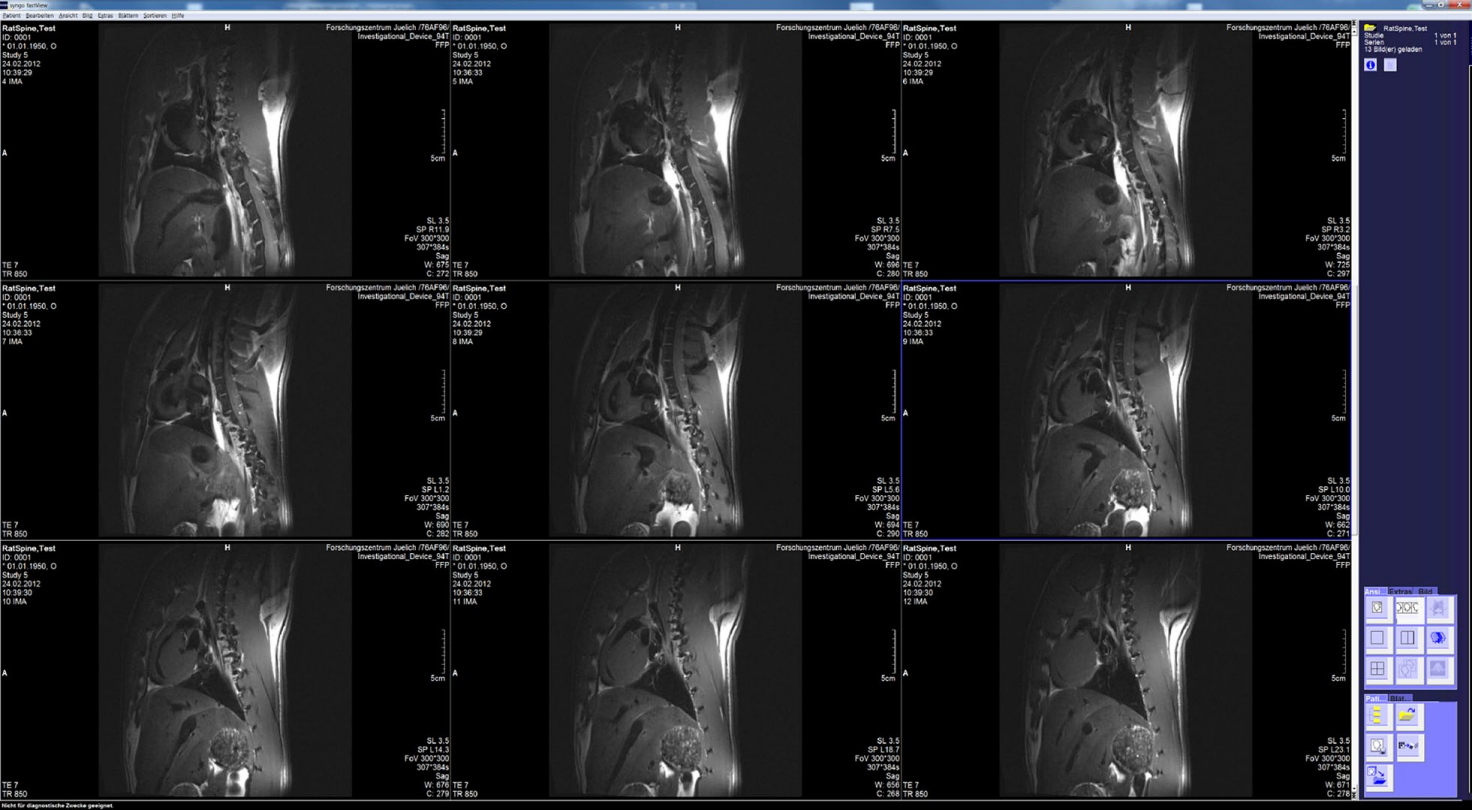
Images of a rat spinal cord acquired on the 9.4 T animal scanner and displayed in the ‘clinical’ user interface

Figure 6 shows sagittal images of a rat spinal cord focusing on the T8/T9 position, which is located directly above the animal’s lung. To eliminate respiratory motion, the acquisitions were gated using a trigger signal derived from the animal monitoring system. Despite the fact that subcutaneous fat yields a hyperintense signal—the fatty tissue is located directly underneath the receiver surface coil-array—the intervertebral discs are clearly visible.

### Performance evaluation: animal system

**Fig. 7:**
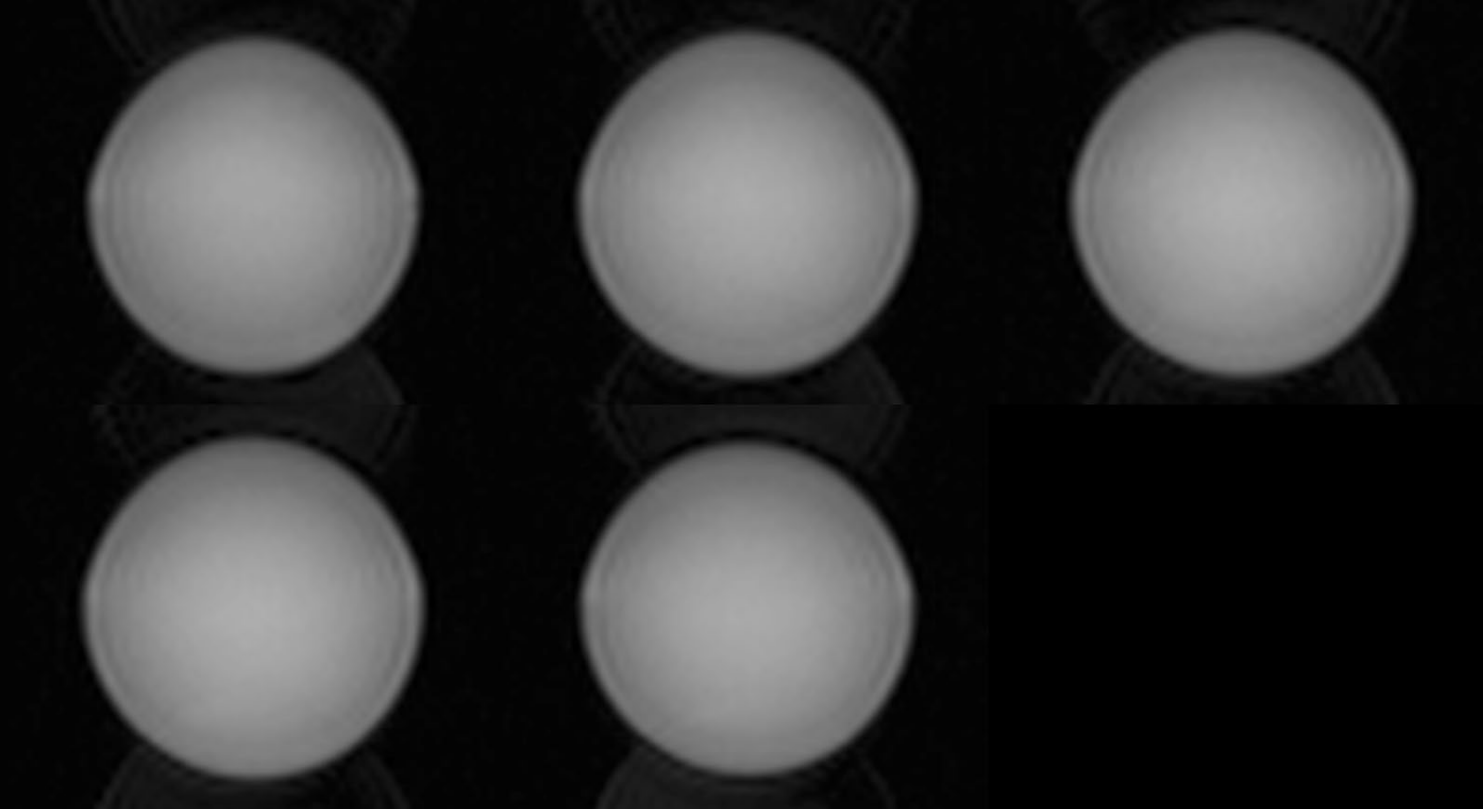
Five slice EPI acquisition of a 40 mm spherical water phantom as a performance evaluation of the gradient system consisting of a clinical amplifier array and an animal gradient insert

Figure 7 shows a five slice EPI acquisition of a spherical phantom. The images show a tolerable degree of geometric distortions and a slight Nyquist N/2 ghosting artefact.

**Fig. 8:**
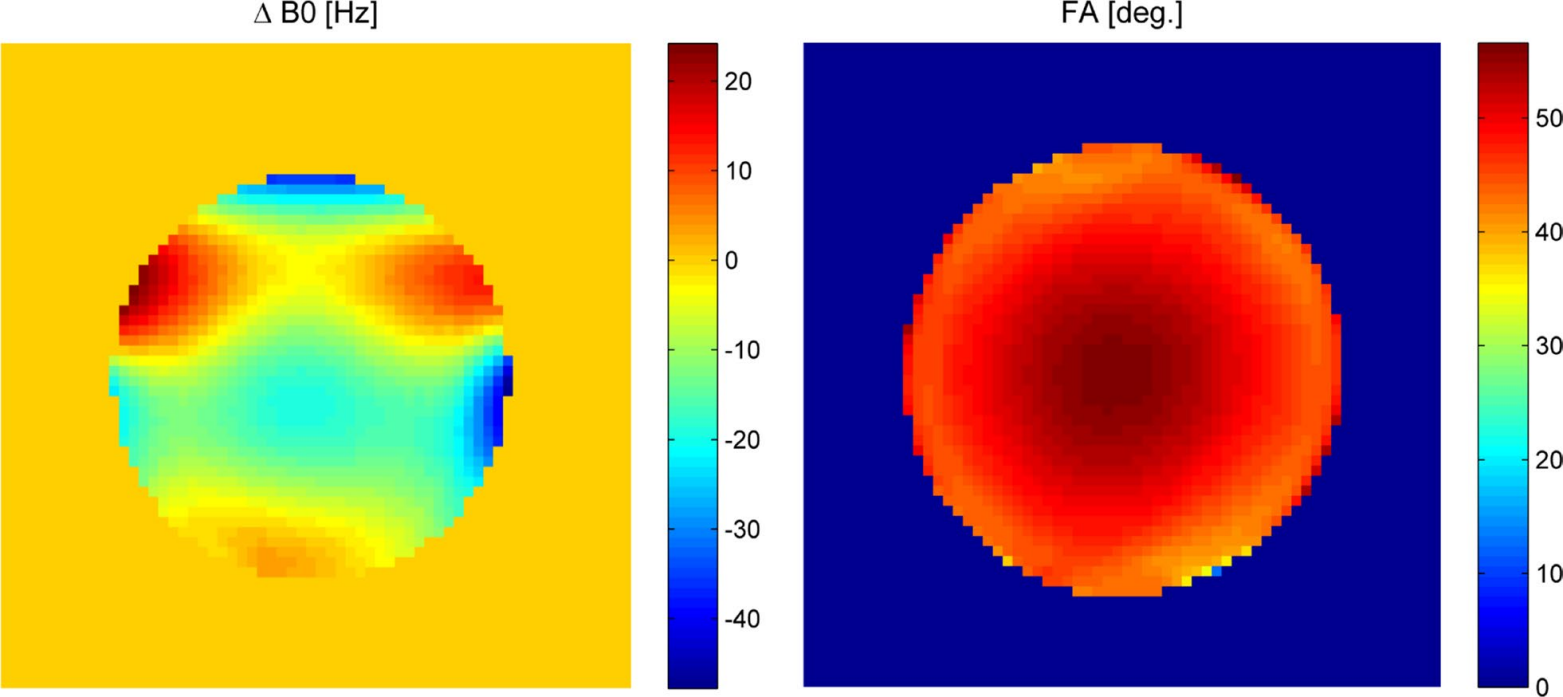
B_0_ homogeneity in Hertz (*left*) and B_1_ homogeneity (*right*) of the central transverse slice in the presented animal scanner

In Fig. 8 central slice homogeneity of the static B_0_ field and the RF B_1_ field are given. Both show reasonable uniformity comparable with those of other commercial animal systems, e.g. compare [21] for a plot of field homogeneities in a 7 T system.

## Discussion

### Translational sequence measurement

**Fig. 9:**
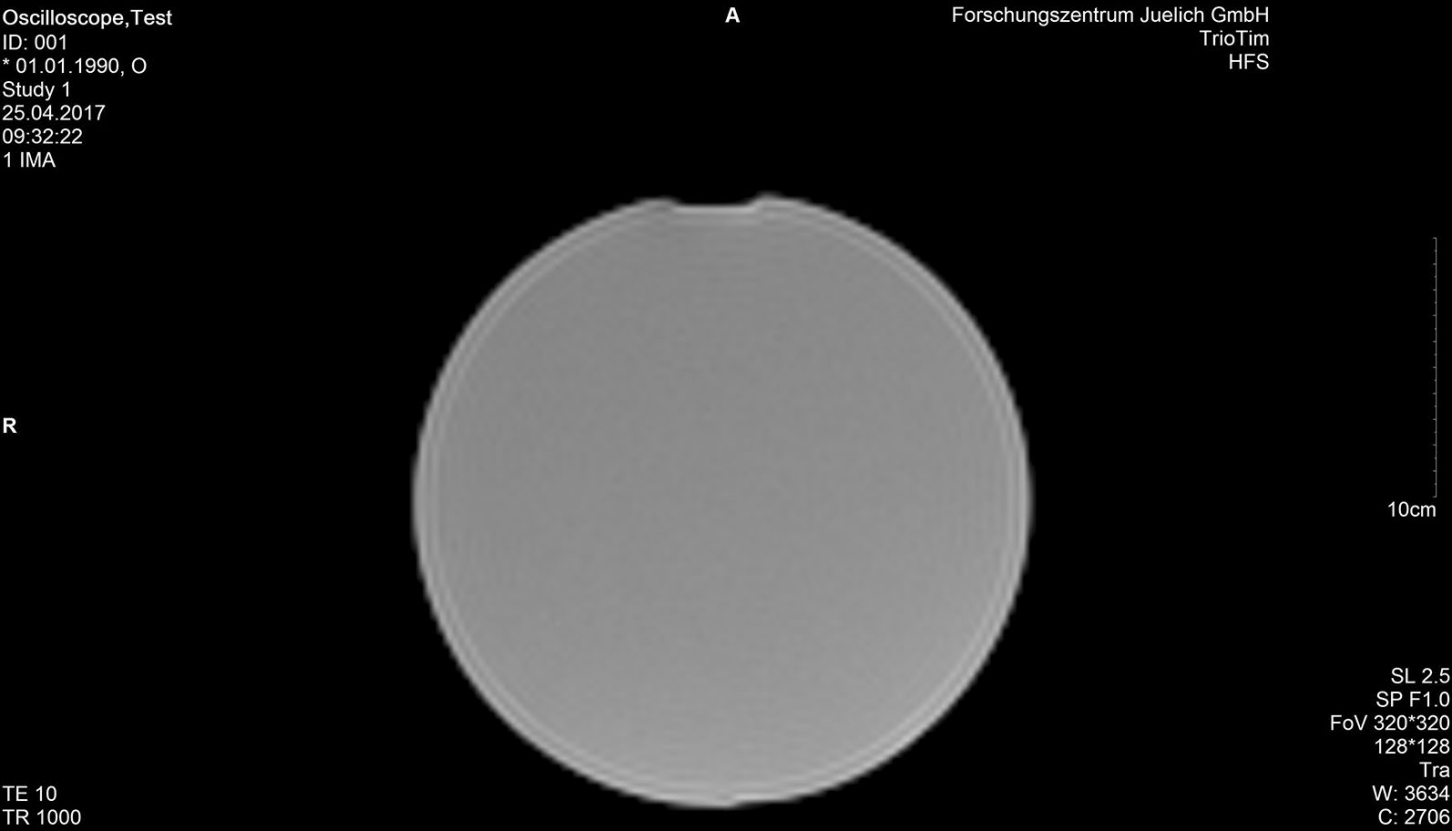
Image of oil-filled phantom without intensity variations

Interestingly, the 3 T image of a water phantom shows some non-uniformity in image intensity, which is not visible in the 9.4 T animal scanner acquisitions. Based on previous experience in high-field measurements, the ring like patterns appear to be wavelength effects caused by constructive and destructive interferences of the RF fields. Consequently, the spin-echo acquisition was repeated using an oil phantom (Bayol oil with a dielectric constant ε_r_ ≈ 2.4 [22]). The acquired image is shown in Fig. 9. The ring-like intensity modulation is no longer present which clearly validates the wavelength assumption. This was reconfirmed by measuring the dielectric constant of both water based phantoms using a dielectric assessment kit (Schmid & Partner Engineering AG, Zurich, Switzerland). The dielectric constant was found to be 79 in both cases and in agreement with results from literature [23]. This results in the larger 3 T phantom having a diameter of approximately 0.63 wavelengths and the smaller 9.4 T animal phantom having a diameter of 0.48 wavelengths. Thus, although operating at a lower frequency, the 3 T phantom is electrically larger than the 9.4 T animal sized phantom.

The gradient waveforms differ slightly due to the different electrical properties of the gradient coils (slew rate and maximum amplitude) as well as different physical FoVs. The same holds for the amplitude of the RF signal as the body-coil of the clinical systems requires significantly higher drive power to achieve the selected flip angle compared to the small diameter but high fill-factor animal system setup.

### Translational imaging result

The images show the expected different contrasts since longitudinal and transversal relaxation times between animals and humans differ, despite measuring at the same main magnetic field strength. An explanation may be due to differences in the structural organisation of human and animal brains [24] resulting in different MR properties. For reference, Table 2 shows numeric values for brain grey and white matter relaxation times at 9.4 T assembled from the literature [25, 26].

**Table 2:** Comparison of relaxation times reported in literature between grey and white matter in humans and animals at 9.4

	Grey matter		White matter	
	T1 (ms)	T2 (ms)	T1 (ms)	T2 (ms)
Human	2002 ± 105	35 ± 3	1425 ± 48	29 ± 2
Animal	2097 ± 68	42 ± 1.6	1660 ± 79	37 ± 2

### General discussion

General applicability and operational reliability was demonstrated with the spinal cord acquisition of a rat in vivo using a turbo-spin echo sequence, which is considered to be a workhorse in clinical imaging protocols [27]. Other studies that have been carried out with the system described here include diffusion measurements [28, 29], phase contrast investigations [30] as well as X-nucleus experiments [15].

An extension of the preclinical scanner currently being implemented is an extension into parallel transmission capability. Driving the available four power amplifiers modules for the ^1^H frequency independently will enable parallel excitation experiments. The envisaged applications include sequence optimization for the 9.4 T human scanner as well as intrinsic parallel-transmit applications, e.g. anatomy-specific excitation to reduce image encoding needs, arbitrary region of interest chemical shift imaging or selective spin tagging. The use of parallel-transmit to mitigate B_1_ inhomogeneities induced by wavelength effects is not envisaged, or required, on the animal 9.4 T scanner.

It should be noted that a 7 T system with a clinical interface has been commercially available but has been discontinued, so that the presented animal MR scanner is the only truly translational platform available at the moment. Also the commercial 7 T system required reprogramming of the software for each release of a new clinical software baseline which could be avoided with the approach presented here. Finally, the authors have already presented X-nuclei capability of their system which has not been feasible with the prior system.

## Conclusions

The successful use of a preclinical MRI scanner integrated with a clinical user interface has been demonstrated. With the translational platform of this machine and standard clinical MRI scanners, multicenter studies become easier as pre-clinical and clinical work can be carried out at different locations but with identical pulse sequences [31]. This is a crucial advantage as multiple sites are now able to share the financial burden of high field systems. Additionally, it facilitates the translation back into today’s clinical field strength, as the platform is also compatible with existing 1.5 T or 3 T installations.

## Abbreviations

AFI: actual flip angle imaging
CAN: controller area network
EPI: echo-planar imaging
FoV: field-of-view
MR: magnetic resonance
MRI: magnetic resonance imaging
PIN: positive–intrinsic–negative
QA: quality assurance
RF: radio frequency
TR: transmit–receive
TX: transmit
